# Immunomodulatory Effects of *Amblyomma variegatum* Saliva on Bovine Cells: Characterization of Cellular Responses and Identification of Molecular Determinants

**DOI:** 10.3389/fcimb.2017.00521

**Published:** 2018-01-04

**Authors:** Valérie Rodrigues, Bernard Fernandez, Arthur Vercoutere, Léo Chamayou, Alexandre Andersen, Oana Vigy, Edith Demettre, Martial Seveno, Rosalie Aprelon, Ken Giraud-Girard, Frédéric Stachurski, Etienne Loire, Nathalie Vachiéry, Philippe Holzmuller

**Affiliations:** ^1^Centre de Coopération Internationale en Recherche Agronomique pour le Développement, UMR ASTRE “Animal, Santé, Territoire, Risques et Ecosystèmes,” Montpellier, France; ^2^ASTRE, Université de Montpellier (I-MUSE), CIRAD, Institut National de la Recherche Agronomique, Montpellier, France; ^3^Institut de Génomique Fonctionnelle, Centre Nationnal de la Recherche Scientifique, Institut National de la Santé et de la Recherche Médicale, Université de Montpellier, Montpellier, France; ^4^BioCampus Montpellier, Centre Nationnal de la Recherche Scientifique, Institut National de la Santé et de la Recherche Médicale, Université de Montpellier, Montpellier, France; ^5^CIRAD, UMR ASTRE, Petit-Bourg, Guadeloupe, France

**Keywords:** *Amblyomma variegatum*, tick saliva, PBMC, immuno-modulation, proteomics

## Abstract

The tropical bont tick, *Amblyomma variegatum*, is a tick species of veterinary importance and is considered as one of major pest of ruminants in Africa and in the Caribbean. It causes direct skin lesions, transmits heartwater, and reactivates bovine dermatophilosis. Tick saliva is reported to affect overall host responses through immunomodulatory and anti-inflammatory molecules, among other bioactive molecules. The general objective of this study was to better understand the role of saliva in interaction between the *Amblyomma* tick and the host using cellular biology approaches and proteomics, and to discuss its impact on disease transmission and/or activation. We first focused on the immuno-modulating effects of semi-fed *A. variegatum* female saliva on bovine peripheral blood mononuclear cells (PBMC) and monocyte-derived macrophages *in vitro*. We analyzed its immuno-suppressive properties by measuring the effect of saliva on PBMC proliferation, and observed a significant decrease in ConA-stimulated PBMC lymphoproliferation. We then studied the effect of saliva on bovine macrophages using flow cytometry to analyze the expression of MHC-II and co-stimulation molecules (CD40, CD80, and CD86) and by measuring the production of nitric oxide (NO) and pro- or anti-inflammatory cytokines. We observed a significant decrease in the expression of MHC-II, CD40, and CD80 molecules, associated with decreased levels of IL-12-p40 and TNF-α and increased level of IL-10, which could explain the saliva-induced modulation of NO. To elucidate these immunomodulatory effects, crude saliva proteins were analyzed using proteomics with an Orbitrap Elite mass spectrometer. Among the 336 proteins identified in *A. variegatum* saliva, we evidenced bioactive molecules exhibiting anti-inflammatory, immuno-modulatory, and anti-oxidant properties (e.g., serpins, phospholipases A2, heme lipoprotein). We also characterized an intriguing ubiquitination complex that could be involved in saliva-induced immune modulation of the host. We propose a model for the interaction between *A. variegatum* saliva and host immune cells that could have an effect during tick feeding by favoring pathogen dissemination or activation by reducing the efficiency of host immune response to the corresponding tick-borne diseases.

## Introduction

*Amblyomma variegatum* is among the most important and widely distributed ticks of tropical livestock, and is the subject of veterinary and public health concerns in Africa and islands in the Indian Ocean and the Caribbean (Stachurski et al., 2010; Bournez et al., 2015). The species is the natural vector of *Ehrlichia ruminantium*, the Rickettsia that causes heartwater in ruminants (Allsopp, 2010), and can also transmit human pathogens including several species of Rickettsia and viruses (Jongejan and Uilenberg, 2004). Moreover, *A. variegatum* has been proven to reactivate the development of dermatophilosis, a skin disease with major economic impacts caused by *Dermatophilus congolensis* (Martinez et al., 1992).

Generally, at the bite site, ticks are able to inhibit pain and itch, inflammation, hemostasis, and wound healing, but also to modulate the host innate and adaptive immune responses, which consequently favor transmission of infectious agents (Šimo et al., 2017). The coevolution between ticks, hosts and pathogens has led to a balance of conflicts and cooperations between the different actors, which mainly benefit the ticks and pathogens. Nevertheless, increased production of antibodies against molecular determinants of tick saliva could also increase protection against pathogen infection (Wade, 2007; Wikel, 2013; de la Fuente et al., 2016). In tick-host interactions, tick saliva is the source of biologically active molecules that target a wide spectrum of host physiological mechanisms, mainly inhibiting host defense reactions to the benefit of the feeding ticks (Kazimírová and Štibrániová, 2013; Stibrániová et al., 2013). The immediate inflammatory response to the skin injury induces rapid infiltration of leukocytes at the tick bite site, where both resident and infiltrated cells (keratinocytes, endothelial cells, mast cells, dendritic cells, macrophages, and lymphocytes) are activated by direct contact with tick saliva (Brossard and Wikel, 2004; Francischetti et al., 2009; Wikel, 2013). Omics analyses have enabled the comprehensive characterization of the molecular determinants of tick saliva, with a functional involvement of these bioactive molecules in host immune suppression (Kotál et al., 2015; Chmelar et al., 2016a,b).

The saliva of *Amblyomma* ticks has been the subject of extensive biochemical studies since the early 2000s (Karim et al., 2004, 2011; Madden et al., 2004; Mulenga et al., 2013; Garcia et al., 2014; Araujo et al., 2016), but only a few have been devoted to the sialome of *A. variegatum*. These studies revealed new families of tick-exclusive proteins (Knizetova et al., 2006; Koh et al., 2007; Ribeiro et al., 2011), some related to suppression of inflammation (Tian et al., 2016).

In the present study, using crude saliva from *A. variegatum* semi-fed females (i.e., at the peak of salivation), our aim was to (i) explore the impact of saliva *in vitro* on bovine immune cells and (ii) determine the molecular immunomodulators. The originality of our approach was to combine cellular experiments, demonstrating modulation of lymphocyte proliferation and macrophage activation, with proteomic analysis of saliva to identify and characterize the immunomodulatory proteins potentially responsible for the observed biological effects. Consequences on host response to infestation by *A. variegatum* and transmission of pathogens are discussed.

## Materials and methods

### *A. variegatum* saliva production

Ten *A. variegatum* female ticks were engorged on naïve creole goats, in accordance with the experimental procedure of the project in animal experimentation approved by the ethics committee Antilles-Guyane (Project Number 69 registered by *Comité National de Réflexion Ethique sur l'Expérimentation Animale*). After 5–7 days, the female ticks were detached at a semi-fed stage and injected in the hemocoele with a Hamilton syringe with 10 μl of a 5% (w/v in PBS) pilocarpine solution to stimulate saliva production. A capillary micropipette placed around the tick hypostome was used to collect the saliva from ticks (duration of salivation about 30 min), and fresh crude saliva was aliquoted and immediately stored at −80°C for further use. For cellular tests, three different pools of saliva were obtained from 10 *A. variegatum* semi-fed female ticks. For proteomic analyses, crude saliva, from either individual ticks' saliva or pools of saliva, was processed directly according the standardized protocol (see below). Protein concentration was measured with a Nanodrop 1000 (ThermoScientific). The concentration of Pilocarpine in the saliva was evaluated using an HPLC-MS/MS method proposed by Ribeiro et al. (2004). Contrary to the pilocarpine concentrations previously reported for *A. americanum* (Ribeiro et al., 2004), we did not detect in our study a concentration of salivary pilocarpine higher than 5 nM, which was set as our pilocarpine control concentration.

### Purification of bovine PBMCs and proliferation assay

Peripheral blood was collected in lithium heparin tubes (BD Biosciences, Le Pont de Claix, France) from three healthy female Jersiaise cattle aged between 4 and 6 years (APAFIS#14442015081310300000). PBMC were isolated by differential centrifugation over Histopaque 1083® (Sigma Aldrich, St. Quentin, France) and washed twice in calcium and magnesium free Hanks balanced salt solution (Life Technologies, Saint Aubin, France). PBMC were suspended at 2.5 × 10^6^ cells/ml in RPMI-1640 culture medium (Life Technologies, Saint Aubin, France) supplemented with 2 mM L-glutamine (Sigma Aldrich, St. Quentin, France), 5 × 10^−5^ M β2-mercaptoethanol (Sigma Aldrich, St. Quentin, France), 50 μg/ml gentamycin (Sigma Aldrich, St. Quentin, France), and 10% heat-inactivated fetal calf serum (Life Technologies, Saint Aubin, France). One hundred microliters of PBMC (2.5 × 10^5^ cells) were distributed in triplicate in the wells of a 96-well microplate and the cells were stimulated with 2.5 μg/ml of Concanavalin A (ConA, Sigma Aldrich, St Quentin, France) either alone (positive control corresponding to 100% of stimulation) or in the presence of *A. variegatum* saliva at concentrations ranging from 0.3 to 37.5 μg/ml for 72 h at 37°C with 5% CO_2_. PBMC were also tested with saliva alone, without ConA stimulation, and with pilocarpine at 5 nM. Cell proliferation was measured using the CyQUANT Direct Cell Proliferation Assay kit (Molecular Probes/ThermoFisher Scientific, Paisley, UK) according to the manufacturer's instructions. Briefly, 100 μl of the 2X detection reagent were added to the cells for a further 60 min of incubation at 37°C, 5% CO_2_. The fluorescence of samples was then read at 480/535 nm using an Enspire Multimode Plate Reader (Perkin Elmer, Courtaboeuf, France). Fluorescence in samples treated with ConA and saliva was compared with fluorescence measured with ConA alone to calculate the relative PBMC activation percentage. Similarly, fluorescence in unstimulated PBMC treated with saliva was compared with fluorescence measured in unstimulated cells. Experiments were performed using cells from three different animals (1973, 9567, and 9906), each of the three saliva batches being tested separately. Data are presented as relative PBMC activation, means of values obtained for the three batches of saliva (for each concentration) in triplicate experiments for each of the three animals.

### Preparation of bovine macrophages and stimulation assay

Immuno-magnetic separation of CD14+ monocytes from PBMC was carried out using anti-human CD14 antibody (TUK4) coated beads (Miltenyi Biotec, Bergish Gladbach, Germany), according to the manufacturer's instructions. Flow cytometry analysis was carried out on the positively selected populations and confirmed that the purity of the population was >95%. Bovine monocytes were suspended in complete IMDM (Life Technologies, Saint Aubin, France) supplemented with 2 mM L-glutamine (Sigma, Saint-Quentin, France), 10% heat-inactivated fetal calf serum (Life Technologies, Saint-Aubin, France), 5 × 10^−5^ M β2-mercaptoethanol (Sigma, Saint-Quentin, France), and 50 μg/ml gentamicin (Life Technologies, Saint-Aubin, France). One hundred microliters of cells at 1.10^6^ cells/ml were seeded into 96-well plates (for NO production) or 500 μl of cells were seeded into 48-well plates (for production of cytokines and analysis of co-stimulation surface markers). Half the culture medium was replaced every 3 days by complete IMDM, supplemented with GM-CSF and INF-γ for NO induction in the 96-well plate. After 7 days of culture, more than 95% of cells had the morphological characteristics of macrophages. At day 7, 100 ng/ml of ultrapure LPS from *E. coli* (InVivoGen, Toulouse, France) was added for 1 h prior to saliva stimulation, alone or in the presence of *A. variegatum* saliva at concentrations ranging from 7.8125 to 250 μg/ml for an additional 24 h at 37°C with 5% CO2. Pilocarpine (5 nM) was also tested. Cells were also tested without LPS pre-stimulation, with the different concentrations of saliva or with 5 nM of pilocarpine. Supernatants were collected from the 96-well plate and NO concentration was immediately determined using the Griess method. Supernatants were collected from the 48-well plate for TNF-α, IL-12, and IL-10 quantification by ELISA, according to the protocols of Hope et al. (2002) and Kwong et al. (2002, 2010). Briefly, 5 μg/ml of capture Mabs CC327, CC301, and CC318 for TNF-α, IL-12, and IL-10, respectively (Bio-Rad AbDSerotec, UK) were used as coating antibodies, cultures were left overnight at +4°C. After washing and blocking with 1 mg/ml of casein (Sigma Aldrich, Saint Quentin, France), cell culture supernatants and serial diluted standards were incubated for 1 h at room temperature. Wells were washed and a 8 μg/ml solution of biotinylated detection Mabs (biotinylated-CC328, -CC326, and -CC320, all from Bio-Rad AbD Serotec, UK) were added for 1 h, detected by Streptavidin-HRP (eBiosciences, France) and revealed with TMB liquid substrate system for ELISA (Sigma Aldrich, France). The reaction was stopped with H_2_SO_4_ 0.5 M and the plates were read at 450 nm. All the results were determined with standard curves obtained with serial dilutions of recombinant proteins (ref. RBOTNFA for TNF-α from Pierce, Rockford, USA; ref. 87464 from AbCam, France for IL-12 and ref. RPO379 for IL-10 from Kingfisher, USA).

Cells in the 48-wells plate were collected and labeled with mouse anti bovine monoclonal antibodies for analysis of co-stimulation surface markers: MHC II (clone J11, kindly provided by ILRI, Kenya), CD40 (clone IL-A156, AbD Serotec, UK), CD80 (clone IL-A159, AbD Serotec, UK), and CD86 (clone IL-A190, AbD Seroteck, UK). Isotypic control was used as non-specific labeling (MCA928, AbD Serotec, UK). Antibodies were revealed using goat anti mouse IgG1-RPE (STAR132PE, AbD Serotec, UK), cells were processed for flow cytometry using a FacsCanto II cytometer (BD Biosciences, USA) and data were analyzed using Diva software (BD Biosciences, USA) for FCS/SSC and FL2 fluorescence intensities. Relative expression in MFI (median of fluorescence intensities) was calculated by comparing stimulated cell (without saliva or with different saliva concentrations) MFI with unstimulated cell MFI. Experiments were performed with cells from three different animals (1973, 9567, and 9906), and tested with a pool of saliva from the three batches.

### Proteomics analysis of *A. variegatum* saliva

*A. variegatum* saliva proteins (10 μg) were separated on SDS-PAGE gels (12% polyacrylamide, Mini-PROTEAN® TGX™ Precast Gels, Bio-Rad, Hercules USA) and stained with Protein Staining Solution (Euromedex, Souffelweyersheim France). Gel lanes were cut into three continuous gel pieces that were treated independently, in order to isolate majority proteins and to allow identification of low quantity proteins. Gel pieces were first destained with 50 mM triethylammonium bicarbonate (TEABC) and three washes in 100% acetonitrile. After protein reduction (with 10 mM dithiothreitol in 50 mM TEABC at 56°C for 45 min) and alkylation (55 mM iodoacetamide TEABC at room temperature for 30 min) proteins were digested in-gel using trypsin (500 ng/band, Gold, Promega, Madison USA) as previously described (Thouvenot et al., 2008). Digest products were dehydrated in a vacuum centrifuge and reduced to 3 μL. The generated peptides were analyzed by nano-flowHPLC–nanoelectrospray ionization using an Orbitrap Elite mass spectrometer (Thermo Fisher Scientific, Waltham USA) coupled to an Ultimate 3000 HPLC (Thermo Fisher Scientific). Desalting and pre-concentration of samples were performed online on a Pepmap® pre-column (0.3 × 10 mm, Dionex). A gradient consisting of 0–40% B for 60 min and 80% B for 15 min (A = 0.1% formic acid, 2% acetonitrile in water; B = 0.1% formic acid in acetonitrile) at 300 nL/min was used to elute peptides from the capillary reverse-phase column (0.075 × 150 mm, Acclaim Pepmap 100®C18, Thermo Fisher Scientific). Eluted peptides were electro-sprayed online at a voltage of 1.9 kV into an Oribtrap Elite mass spectrometer. A cycle of one full-scan mass spectrum (400–2,000 m/z) at a resolution of 120,000 (at 400 m/z), followed by 20 data-dependent MS/MS spectra was repeated continuously throughout the nanoLC separation. All MS/MS spectra were recorded using normalized collision energy (33%, activation Q 0.25 and activation time 10 ms) with an isolation window of 2 m/z. Data were acquired using Xcalibur software (v 2.2). For all full scan measurements with the Orbitrap detector a lock-mass ion from ambient air (m/z 445.120024) was used as an internal calibrant as described in Olsen et al. (2005). MS data were analyzed using the MaxQuant software package (v 1.5.0.0) as described by Cox and Mann (2008). Tandem mass spectra (MS/MS) were searched for using the Andromeda search engine (Cox et al., 2011) against the UniProtKB database (release 2015_03) for the *A. variegatum* (AMBVA) taxonomy (753 entries), Amblyomma “all species” taxonomy (22,429 entries), and the UniProtKB proteome UP000001555 database for *Ixodes scapularis* (20,473 entries) using the following parameters: enzyme specificity was set as Trypsin/P, and a maximum of two missed cleavages and a mass tolerance of 0.5 Da for fragment ion were applied. A second database of known contaminants provided with the MaxQuant suite was also used. The “match between runs” option was checked. Oxidation (M) was specified as variable modification and carbamidomethyl (C) as fixed modification. Database searches were performed with a mass tolerance of 20 ppm for precursor ion for mass calibration, and with a 4.5 ppm tolerance after calibration. The maximum false peptide and protein discovery rate was set at 0.01. The MaxQuant software generates several output files that contain information about the peptides and proteins identified. The “proteinGroups.txt” file is dedicated to identified proteins: each single row collapses all proteins that cannot be distinguished based on identified peptides into protein groups. An in-house bioinformatics tool was developed to automatically select a representative protein ID in each protein group. First, proteins with the most identified peptides were isolated in a group called “match group” [proteins from the “Protein IDs” column with the maximum number of “peptides counts (all)”]. For 35% of the remaining match groups in which more than one protein ID existed, the “leading” protein was chosen as the best annotated protein according to the number of gene ontology annotations (retrieval from UniProtKB March 20, 2015) and/or given the following species order preference: *A. variegatum* > *A. maculatum* > *A. cajennense* > *A. parvum* > *A. americanum* > *A brasiliense* > *A. triste* > *A. geayi* > *A. scutatum* > *A. rotundatum*. The mass spectrometry data along with the identification results were deposited in the ProteomeXchange Consortium via the PRIDE (Martens et al., 2005) partner repository with the dataset identifier PXD007821.

### Bioinformatics analysis of proteomics data

We used several web servers to investigate the biochemical properties of the identified proteins.

The ProtFun 2.2 server produces *ab initio* predictions of protein function from sequences. The method queries a large number of other feature prediction servers to obtain information on various post-translational and localizational aspects of the protein, which are integrated in final predictions of the cellular role, enzyme class (if any), and selected gene ontology (GO) categories of the submitted sequence (Jensen et al., 2002, 2003).

Propsearch server is designed to find the putative protein family if querying a new sequence using alignment methods has failed. Disregarding the order of amino acid residues in a sequence, Propsearch uses the amino acid composition instead. In addition, other properties like molecular weight, bulky residue content, small residue content, average hydrophobicity, average charge a.s.o. and the content of selected dipeptide-groups are also calculated from the sequence. A total of 144 such properties are weighted individually and used as query vectors. The weights have been trained on a set of protein families with known structures, using a genetic algorithm. Sequences in the database are also transformed into vectors, and the Euclidian distance between the query and database sequences is calculated. Distances are rank ordered, and sequences with lowest distance are reported on top (Hobohm and Sander, 1995). We used Propsearch first to better characterize proteins identified as “Putative Uncharacterized Protein” and second to improve the potential functional information on the identified proteins. In our analytical strategy, we conserved only data for proteins with Euclidian distances between 0.0 and 1.3 (Reliability 99.9%) and between 1.3 and 7.5 (Reliability 99.6%).

### Statistical tests

Graphs and linear modeling were performed with R version 3.4.1 (R Core Team, 2013) and the tidyverse package (v 1.1.1). Datasets and figures along with the script are available at: https://github.com/loire/amblyoma_paper.

## Results

### Inhibition of ConA-induced PBMC proliferation by *A. variegatum* saliva

**Figure 1:**
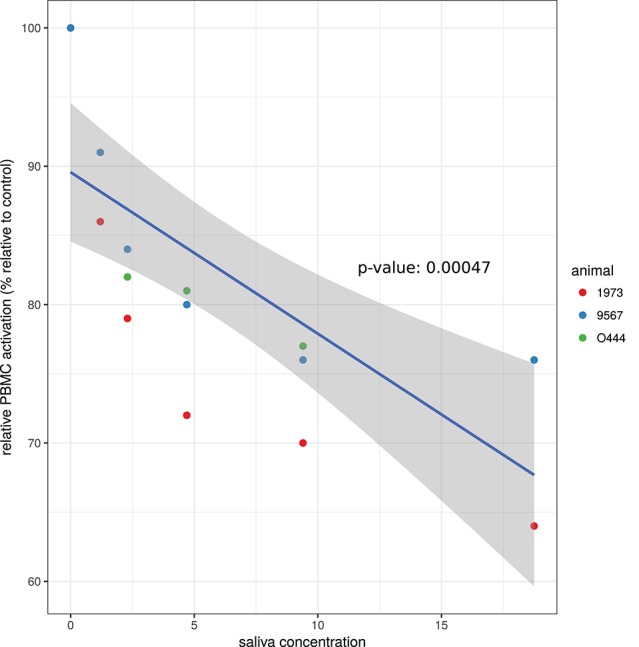
Effect of *A. variegatum* saliva on ConA-induced proliferation of bovine PBMC. Bovine PBMC were stimulated with ConA alone or with different concentrations of *A. variegatum* saliva for 72 h. PBMC responses of cells in contact with ConA and saliva were compared to PBMC in contact with ConA alone (control) to calculate “relative PBMC activation.” Data are means of triplicate experiments, for each batch of saliva for the three animals tested. Blue lines and shading show respectively the linear regression fit and the 0.95 confidence interval. The *p*-value of the model is indicated on the panel.

To investigate the effect of *A. variegatum* on PBMC proliferation, cells were stimulated with ConA alone (positive control for PBMC activation) or with various concentrations of saliva for 72 h, and the relative PBMC activation for each saliva concentration vs. no saliva (positive control) was calculated. As shown in Figure 1, the relative PBMC activation decreased in a dose-dependent manner when cells were in contact with *A. variegatum* saliva. A concentration of 1.2 μg/ml of saliva reduced the ConA-induced proliferation of PBMC by 9–13%, depending on the animal from which the cells originated (Figure 1). The decrease in proliferation was linear depending on the concentration of the saliva, and reached 24–36% for 18.75 μg/ml of saliva (Figure 1). Saliva concentrations of <1.2 μg/ml was tested and did not result in statistically significant inhibition of ConA-induced PBMC proliferation, whereas levels of inhibition for higher concentrations (>18.75 μg/ml) were similar to those for 18.75 μg/ml (data not shown). Unstimulated PBMC incubated with saliva did not show any proliferation and pilocarpine control had no effect on ConA-induced PBMC proliferation (data not shown).

### Modification of LPS pre-stimulated macrophage activation by *A. variegatum* saliva

**Figure 2:**
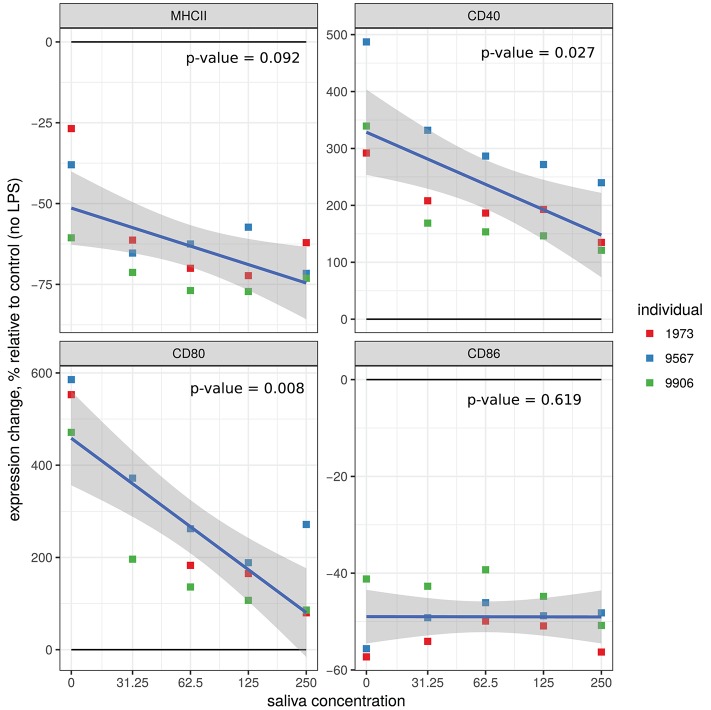
Effect of *A. variegatum* saliva on the expression of surface markers on LPS-stimulated bovine macrophages. Bovine blood monocyte-derived macrophages were pre-stimulated for 1 h with LPS, and then for an additional 24 h with different concentrations of *A. variegatum* saliva. Cells were collected and stained for surface markers. Expression levels (mean of fluorescence intensity, MFI) of MHC II, CD40, CD80, and CD86 markers on macrophages compared with unstimulated control cells were measured by flow cytometry. Data are means of triplicate experiments, for a pool of saliva batches for the three animals tested. Blue lines and shading show the linear regression fit and the 0.95 confidence interval, respectively. The *p*-value of the model is indicated on the panel.

We tested the effects of various concentrations of *A. variegatum* saliva on MHC II and co-stimulation molecules (CD40, CD80, and CD86) on LPS pre-stimulated macrophages. As shown in Figure 2, in all conditions tested, *A. variegatum* saliva had a biological effect on the expression of co-stimulation surface markers in LPS pre-stimulated macrophages, compared to unstimulated cells (no LPS). Saliva enhanced the LPS-induced down regulation of macrophages MHC II from 23 to 62%, from 39 to 72%, or from 60 to 75% depending on both the saliva concentration and the animal (overall *p* = 0.092, specific *p* < 0.05), and decreased the LPS-induced up regulation of both CD40 and CD80 in a dose-dependent manner (Figure 2). The saliva had no obvious effect on LPS-induced down regulation of CD86, which was already strongly decreased by LPS alone (Figure 2). The three independent animals gave similar profiles with individual variability in the level of expression of the co-stimulatory surface markers but, as shown in Figure 2, the trend curve shows that the individual variations all followed the same pattern. The effect of *A. variegatum* saliva alone on macrophages, without pre-stimulation with LPS, was less striking, with marked individual variability between animals: MHC II and CD86 were down regulated in a dose-dependent manner, CD40 and CD80 were up regulated in two out of three animals with no dose-dependence, CD86 was down regulated in a dose-dependent manner (Supplementary Figure 1). Percent expression of CD40 and CD80 induced by saliva alone was 10 times lower than that with LPS pre-stimulation (Supplementary Figure 1). Macrophages derived from animal n° 1973 exhibited a difference MHC II levels depending on the concentration of the saliva compared to the other animals: expression increased for 31.25 μg/ml of saliva, then decreased for 62.5 μg/ml, increased slightly for 125 μg/ml and decreased slightly for 250 μg/ml. Same kind of inverted fluctuations were observed with animal n° 9906 for CD80 and animal n° 9567 for CD86 (Supplementary Figure 1).

**Figure 3:**
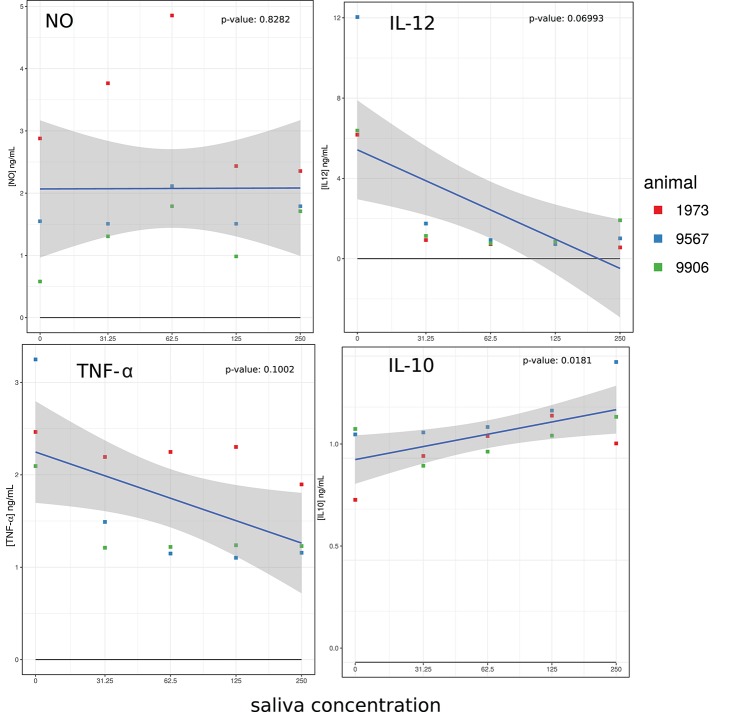
Effect of *A. variegatum* saliva on the production of NO and cytokines by LPS-stimulated bovine macrophages. Bovine blood monocyte-derived macrophages were pre-stimulated for 1 h with LPS, and then for an additional 24 h with different concentrations of *A. variegatum* saliva. Cell culture supernatants were collected and NO production was titrated by the Griess method, IL-10, IL-12, and TNF-α production were titrated by ELISA. Data are means of triplicate experiments, for a pool of saliva batches for the three animals tested. Blue lines and shading show the linear regression fit and the 0.95 confidence interval, respectively. The *p*-value of the model is indicated on the panel.

As shown in Figure 3, LPS pre-stimulated macrophages produced NO and production was increased by adding up to 62.5 μg/ml of *A. variegatum* saliva, but decreased at higher saliva concentrations (125 and 250 μg/ml). The production of cytokines was also affected: IL-12 and TNF-α levels decreased dramatically, from a concentration of 31.25 μg/ml of saliva, except for animal n° 1973, in which a small dose-dependent decrease in TNF-α was observed (Figure 3). In contrast, the IL-10 level was increased by saliva in a dose-dependent manner (Figure 3). The three independent animals exhibited similar NO profiles and cytokine production, with individual variability in the levels reached but, like for co-stimulation surface markers, the trend curve showed that the individual variations all followed the same pattern. The effect of saliva alone on unstimulated cells resulted in very low levels of cytokine production, with increased levels of IL-10 up to 125 μg/ml of saliva, increased level of IL-12 up to 31.25 μg/ml then decreased IL-12 in a dose dependent manner, slightly decreased level of TNF-α in a dose-dependent manner (Supplementary Figure 2). Unstimulated cells did not produce NO in the presence of saliva (data not shown).

### Molecular characterization of *A. variegatum* saliva

Proteomics analysis of *A. variegatum* crude saliva led to the identification of 336 proteins, with no significant qualitative and quantitative variation between individuals (PRIDE PXD007821), allowing us to use pools of saliva for both cell biology assays and molecular characterization. The protein sequence data, consisting of the *Amblyomma* taxonomy (22,429 entries) and the *I. scapularis* proteome (UP000001555, 20,473 entries), were retrieved from UniProtKB (release 2015_03) for identification purposes. Among the 336 proteins identified, only 58 (17.3%) *A. variegatum* specific proteins were identified, knowing that the UniProtKB *A. variegatum* specific/particular database contained 753 entries (UniProtKB release 2015_03). Other identified proteins were divided into 236 *Amblyomma* (70.2%) and 42 *I. scapularis* (12.5%), which is the only ixodid tick species with a reference proteome in UniProtKB. It should be noted that 58 out of the 336 (17.3%) proteins identified were described as “Uncharacterized protein.”

**Figure 4:**
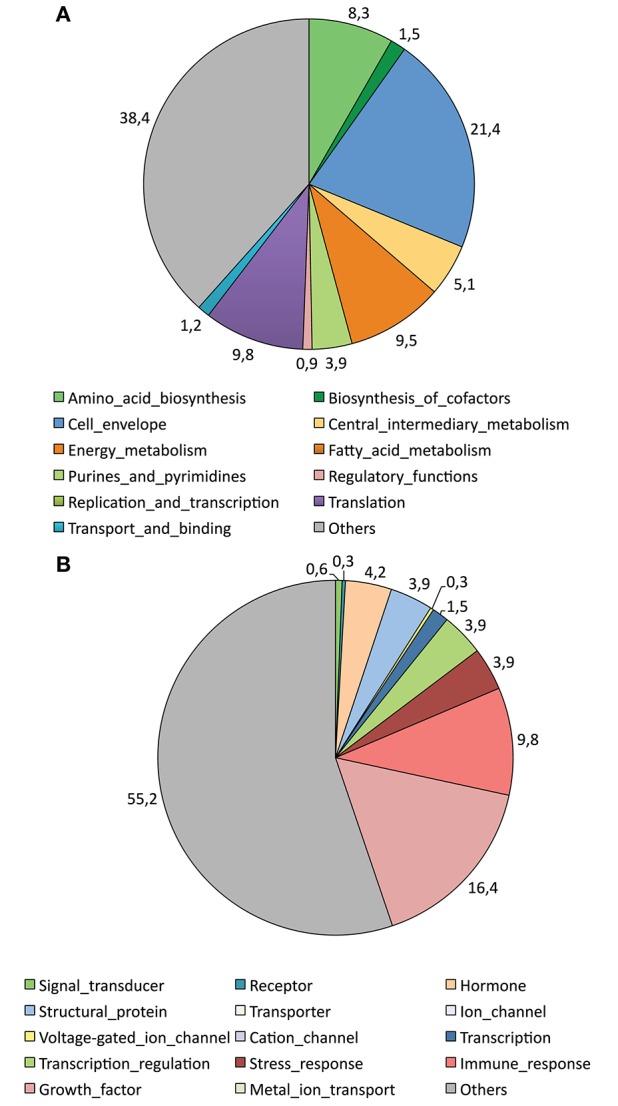
Graphic representation of cellular role **(A)** and selected gene ontology (GO) categories **(B)** attributed to proteins identified in the saliva of *A. variegatum* by the ProtFun 2.2 Server (http://www.cbs.dtu.dk/services/ProtFun/). The function prediction server produces *ab initio* predictions of protein function from sequences by querying a large number of other feature prediction servers to obtain information on different post-translational and localizational aspects of the protein, which are integrated into final predictions of the cellular role, enzyme class (if any), and selected gene ontology categories of the sequence submitted.

Analysis of the nature of the identified proteins by the ProtFun 2.2 server indicated that 239 out of 336 (71.1%) of the proteins identified in *A. variegatum* saliva were enzymes. The predicted enzymes were further classified in four out of six different classes (51 Lyases, 25 Ligases, 12 Isomerases, 2 Hydrolases with no Oxidoreductase, and no Transferase), but 149 remained unclassified. Moreover, Figure 4A shows the classification of the identified proteins in 12 different functional categories by the ProtFun 2.2 server, the most widely represented for *A. variegatum* saliva being Cell envelope (21.4%), Translation (9.8%), Energy metabolism (9.5%), and Amino acid biosynthesis (8.3%). Nevertheless, 38.4% of the identified proteins were not classified in functional categories (Figure 4A). Figure 4B shows the prediction scores for 14 gene ontology (GO) categories, the most frequently represented for *A. variegatum* saliva being Growth factor (16.4%) and Immune response (9.8%). Like the functional categories, 55.2% of the identified proteins were not classified in the proposed GO categories.

In order to link the molecular data with the biological data obtained in the cell experiments, we focused on the characterization of *A. variegatum* saliva proteins with immunomodulatory properties already described in the literature in other tick species. We found 89 out of 336 (26.5%) with a potential role in the modulation of the host immune response. Among the immunomodulatory proteins, Table 1 shows a classification into different groups of salivary proteins with immunomodulatory functions (linked with the observed effect of *A. variegatum* saliva on the host immune cells) as follows: immunogenic proteins including Da-p36 immunosuppressant and Antigen-5 family member (CAP superfamily of proteins), proteases inhibitors including serpins and other serine and systeine protease inhibitors, proteases including serine proteases/serine carboxypeptidases/cathepsin-like cysteine proteases, ferritin and hemelipoproteins, calcium binding proteins, fatty acid, and histamine binding protein, nucleic acid binding proteins, anti-oxidant enzymes, heat shock proteins.

**Table 1:** Potential immunomodulatory proteins identified in *A. variegatum* saliva.

**UniProt protein IDs (Leading protein)**	**Description**	**Organism**	**Number of proteins**	**Peptides**	**Unique peptides**	**Sequence coverage [%]**	**Mol. weight [kDa]**
**IMMUNOGENIC PROTEINS**							
Q75PV7	Protein related to Da-p36	*A. variegatum*	1	6	6	29.7	27.443
F0J8E1	Antigen-5 family member (Fragment)	*A.variegatum*	1	2	2	32.7	12.308
**PROTEASE INHIBITORS INCLUDING**							
**Serpins**							
F0J9G9	Serpin (Fragment)	*A. variegatum*	6	11	9	59.3	23.07
F0J8P8	Serpin (Fragment)	*A. variegatum*	1	5	5	57.1	17.759
F0J8R0	Serpin (Fragment)	*A. variegatum*	1	2	1	29.1	17.208
A0A023GPF9	Putative tick serpins 14	*A. triste*	6	6	6	14.7	44.703
A0A023GN51	Putative tick serpins 13	*A. triste*	6	5	1	12.1	42.841
A0A023GP80	Putative tick serpins 27	*A. triste*	2	3	2	7.6	43.538
A0A023FPT3	Putative tick serpins 7 (Fragment)	*A. cajennense*	1	2	2	7.5	35.74
A0A023GGH1	Putative tick serpins 33 (Fragment)	*A. triste*	1	2	2	8.5	26.402
A0A023FUL1	Putative tick serpins 12 (Fragment)	*A. cajennense*	3	2	2	7.8	46.875
A0A023GPF5	Putative tick serpins 13	*A. triste*	2	2	1	9.3	42.797
G3MLB1	Putative uncharacterized protein (tick serpins 27)	*A. maculatum*	15	3	1	9.6	43.103
**Other serine protease inhibitors**							
F0J9Y3	Kazal-type serine protease inhibitor domain protein	*A. variegatum*	4	3	3	20	26.71
A0A023GE28	Putative serine protease inhibitor *I. scapularis* serine protease inhibitor (Fragment)	*A. triste*	2	6	6	18.5	46.98
A0A023GE28	Putative serine protease inhibitor *I. scapularis* serine protease inhibitor (Fragment)	*A. triste*	2	6	6	18.5	46.98
A0A023FQ10	Putative serine protease inhibitor *I. scapularis* serine protease inhibitor	*A. cajennense*	1	2	2	5.1	44.715
A0A023GPF6	Putative serine proteinase inhibitor	*A. triste*	2	2	1	8.4	43.504
**Cysteine protease inhibitors**							
A0A023FR27	Putative thyropin	*A. cajennense*	6	2	2	12.4	22.395
F0JA40	Putative thyropin	*A. variegatum*	7	3	3	12.7	22.532
**PROTEASES INCLUDING**							
**Serine proteases**							
F0J9J5	Trypsin-like serine protease (Fragment)	*A. variegatum*	1	6	6	41.1	21.867
F0J9E7	Trypsin-like serine protease (Fragment)	*A. variegatum*	3	6	4	48.9	19.618
**Serine carboxypeptidases**							
A0A023GNB7	Putative serine carboxypeptidase lysosomal cathepsin a	*A. triste*	1	1	1	5.2	53.097
**Cathepsin-like cysteine proteases**							
F0JA27	Cathepsin L-like cysteine proteinase B	*A. variegatum*	8	10	9	36.8	37.624
F0J903	Cysteine proteinase cathepsin L (Fragment)	*A. variegatum*	2	5	5	38.6	23.017
G3MHB0	Putative uncharacterized protein (Fragment) (peptidase C1 family; cathepsin l-like cysteine proteinase b)	*A. maculatum*	3	2	1	11.5	38.867
**FERRITIN AND HEMELIPOPROTEINS**							
G3MPJ3	Ferritin	*A. maculatum*	2	3	3	19.5	22.296
F0J8W4	Heme lipoprotein (Fragment)	*A. variegatum*	2	33	22	63.5	50.007
F0J9N1	Heme lipoprotein (Fragment)	*A. variegatum*	1	20	18	67.4	27.005
F0J9N8	Hemelipoglycoprotein (Fragment)	*A. variegatum*	1	14	14	48.2	24.787
F0J9G0	Heme lipoprotein (Fragment)	*A. variegatum*	1	11	8	95.4	14.828
A0MVX0	Heme lipoprotein	*A. americanum*	1	31	5	16.3	177.04
B7Q406	Hemelipoglycoprotein. putative	*I. scapularis*	2	3	1	1.7	177.65
B7QIL0	Hemelipoglycoprotein. putative (Fragment)	*I. scapularis*	1	2	1	1.3	172.44
**CALCIUM BINDING PROTEINS**							
F0JA25	Calreticulin	*A. variegatum*	1	4	1	16.3	47.318
Q64KA0	Calreticulin	*A. brasiliense*	17	4	1	16.3	47.241
G3MK71	Calponin	*A. maculatum*	6	3	3	24.7	17.614
**FATTY ACID AND HISTAMINE BINDING PROTEIN**							
A0A023G6C8	Putative lipocal-1 1	*A. triste*	1	1	1	8.5	24.71
A0A023GBM1	Putative phospholipase a2 *I. scapularis* phospholipase a2	*A. triste*	2	4	4	11.4	44.79
**NUCLEIC ACID BINDING PROTEINS**							
G3MSL8	Putative uncharacterized protein (Putative gtp-binding protein)	*A. maculatum*	10	2	2	12.4	24.689
G3MRA1	Putative uncharacterized protein (gtp-binding protein)	*A. maculatum*	4	1	1	6	22.968
A0A023FGS1	Putative zinc-binding protein of the histidine triad hit family	*A. cajennense*	3	1	1	11.1	13.53
**ANTI-OXYDANT ENZYMES**							
A0A023FIC2	Putative glutathione s-transferase mu class *Rhipicephalus annulatus* glutathione s-transferase	*A. cajennense*	7	6	6	29.1	25.713
A0A023GJM3	Putative glutathione s-transferase	*A. triste*	2	2	2	14.7	24.394
A0A023FXR2	Putative glutathione s-transferase *I. scapularis* glutathione s-transferase	*A. parvum*	5	2	2	10.4	26.241
A0A023FFS7	Putative glutathione s-transferase *I. scapularis* glutathione s-transferase	*A. cajennense*	1	1	1	5.5	24.303
A0A023FQ81	Putative glutathione s-transferase *I. scapularis* glutathione s-transferase (Fragment)	*A. cajennense*	4	1	1	8.9	23.244
A0A023G5I8	Putative glutathione s-transferase *I. scapularis* glutathione s-transferase (Fragment)	*A. triste*	4	1	1	8.6	27.412
B7PDV0	Glutathione S-transferase. putative	*I. scapularis*	4	1	1	12.2	8.6628
G3MMK0	Putative uncharacterized protein (glutathione s-transferase *I. scapularis* glutathione s-transferase)	*A. maculatum*	2	1	1	5.9	26.159
A0SHR2	Protein disulfide-isomerase	*A. variegatum*	8	3	3	7.2	54.923
A0A023FZW2	Protein disulfide-isomerase	*A. parvum*	5	4	1	9.2	57.252
A0A023FNM6	Putative alkyl hydroperoxide reductase thiol specific antioxidant (Fragment)	*A. cajennense*	2	2	2	10.7	23.542
A0A023FHT1	Putative thioredoxin peroxidase	*A. cajennense*	14	4	4	24	20.494
A0A023FDL5	Putative selenoprotein w 2a	*A. cajennense*	1	1	1	22.1	8.6959
F0J9H3	Peroxidasin (Fragment)	*A. variegatum*	1	1	1	8.1	27.07
A0A023FTX2	Superoxide dismutase [Cu-Zn]	*A. parvum*	1	1	1	9.7	15.744
**HEAT SHOCK PROTEINS**							
A0A023GNY6	Putative heat shock protein (Fragment)	*A. triste*	11	16	13	35.6	73.124
A0A023GP15	Putative heat shock protein 90	*A. triste*	9	7	2	13.5	84.001
A0A023GLJ3	Putative heat shock protein 70	*A. triste*	7	5	2	10.5	73.014
A0A023FRH0	Putative heat shock protein	*A. cajennense*	1	1	1	5.7	21.73
G3MF27	Putative uncharacterized protein (heat shock 70 kDa protein 5)	*A. maculatum*	7	4	3	8	69.078
G3MF42	Putative uncharacterized protein (heat shock protein)	*A. maculatum*	2	1	1	7.8	21.722

Twelve proteins were identified as ubiquitin with different protein IDs from *Amblyomma* and *Ixodes* taxa, of which 2 (F0J9P3, F0J9K1) specifically in *A. variegatum* (Table 2). Using the Propsearch server, we were able to characterize 6 saliva proteins that exhibited properties of ubiquitin-activating enzyme E1 ligase, with respective Euclidian distances, giving a reliability of 99.6% (Table 2). By contrast, we found ubiquitin-conjugating enzyme E2 ligase with Euclidian distances giving a percent reliability below the threshold fixed in our analytical approach (from 68 to 94%), and no ubiquitin-ligating enzyme E3 ligase (Table 2). However, we found 8 potential de-ubiquitinating enzymes, mainly ubiquitin carboxyl-terminal hydrolases, suggesting a complete and potentially functional ubiquitination system in *A. variegatum* saliva (Table 2). We found the missing link due to the identification of an alpha spectrin (Table 2), which has the particularity of being described as a potential chimeric E2/E3 ubiquitin conjugating/ligating enzyme (Goodman et al., 2015).

**Table 2:** Potential ubiquitination complex identified in *A. variegatum* saliva.

**UniProt protein IDs (Leading protein)**	**Description**	**Organism**	**Number of proteins**	**Peptides**	**Unique peptides**	**Sequence coverage [%]**	**Mol. weight [kDa]**	**Propsearch distance (reliability)**	**Propsearch putative protein family**
**UBIQUITIN**									
F0J9K1	Ubiquitin (Fragment)	*A. variegatum*	12	2	2	29.8	9.4959	–	–
**E1 UBIQUITIN LIGASE**									
A0A023GDS5	Putative microtubule associated complex (Fragment)	*A. triste*	2	12	9	14.1	117.06	5.38 (99.6%)	Ubiquitin-activating enzyme E1 (A1S9 protein).
A0A023G500	Putative nidogen (Fragment)	*A. triste*	1	1	1	1.2	124.2	6.21 (99.6%)	Ubiquitin-activating enzyme E1 homolog (D8)
B7QMC8	Alpha-macroglobulin. putative (Fragment)	*I. scapularis*	1	8	1	7	121.21	4.83–5.37 (99.6%)	Ubiquitin-activating enzyme E1
B7PN44	Netrin-4. putative	*I. scapularis*	1	2	2	5.1	41.593	7.21 (99.6%)	Ubiquitin-activating enzyme E1 X (Fragment)
B7PQH0	Hemolectin. putative (Fragment)	*I. scapularis*	1	2	2	2.4	115.88	5.66–5.67 (99.6%)	Ubiquitin-activating enzyme E1
A0A023GPI0	Uncharacterized protein (Fragment)	*A. triste*	3	28	1	32.3	131.52	4.90–5.42 (99.6%)	Ubiquitin-activating enzyme E1
**E2 UBIQUITIN LIGASE**									
B5M727	Alpha-2-macroglobulin (Fragment)	*A. americanum*	2	5	2	42.6	19.026	8.69–10.05 (94% > x > 80%)	Ubiquitin-conjugating enzyme E2-24 kDa (EC 6.3.2.19)
A0A023G356	Putative secreted protein (Fragment)	*A. triste*	1	3	3	12.2	21.619	11.17 (80%)	Ubiquitin-conjugating enzyme E2-24 kDa (EC 6.3.2.19)
A0A023FDX3	Putative I. scapularis glutathione peroxidase (Fragment)	*A. cajennense*	1	1	1	12.8	10.918	11.13–12.43 (80% > x > 68%)	Ubiquitin-conjugating enzyme variant MMS2 homolog (UEV MMS2)
G3MNL6	Putative uncharacterized protein (alpha crystallins?)	*A. maculatum*	2	1	1	13.1	19.912	9.69 (87%)	Ubiquitin-conjugating enzyme E2-24 kDa (EC 6.3.2.19)
**E2/E3 UBIQUITIN LIGASE**									
B7P1U8	Spectrin alpha chain. putative	*I. scapularis*	4	4	4	2	272.3	–	–
**DE-UBIQUITINATING ENZYME**									
A0A023GME3	Putative vitellogenin-1 (Fragment)	*A. triste*	1	38	10	28.4	153.63	6.12–7.44 (99.6%)	Ubiquitin carboxyl-terminal hydrolase 15 (EC 3.1.2.15)
A0A023GMC7	Putative vitellogenin-1 (Fragment)	*A. triste*	2	34	6	30.6	123.4	6.42–6.80 (99.6%)	Ubiquitin carboxyl-terminal hydrolase 15 (EC 3.1.2.15)
A0A023GP74	Putative titin (Fragment)	*A. triste*	2	21	15	10.4	389.34	7.13 (99.6%)	Probable ubiquitin carboxyl-terminal hydrolase FAF (EC 3.1.2.15)
A0A023G0F9	Putative peroxinectin I. scapularis peroxinectin (Fragment)	*A. parvum*	1	16	3	32.1	87.425	5.42 (99.6%)	Ubiquitin carboxyl-terminal hydrolase 20 (EC 3.1.2.15)
A0A023GCH5	Putative neurexin iii-alpha (Fragment)	*A. triste*	1	9	9	8.4	158.55	5.72 (99.6%)	Ubiquitin carboxyl-terminal hydrolase 19 (EC 3.1.2.15)
A0A023GLZ9	Putative extracellular matrix glycoprotein laminin subunit beta (Fragment)	*A. triste*	1	9	4	15.7	92.371	5.25 (99.6%)	Ubiquitin carboxyl-terminal hydrolase 20 (EC 3.1.2.15)
B7P403	Type II transmembrane protein. putative	*I. scapularis*	1	6	6	2.6	319.6	5.79 (99.6%)	Probable ubiquitin carboxyl-terminal hydrolase FAF (EC 3.1.2.15
B7PUM7	Peroxinectin. putative	*I. scapularis*	1	4	1	6.7	90.84	5.18 (99.6%)	Ubiquitin carboxyl-terminal hydrolase 20 (EC 3.1.2.15)

Using Propsearch server, we were able to complete the characterization of *A. variegatum* saliva proteins with immunomodulatory properties, by classifying five additional proteins associated with modulation of the host immune response as follows (Table 3): a GTP-binding protein (nucleic-acid binding proteins), a superoxide dismutase and a D-dopachrome tautomerase (anti-oxidant enzymes), a 60S ribosomal protein L3 (ribosomal proteins), and another alpha spectrin (Ubiquitin complex).

**Table 3:** Propsearch-defined potential immunomodulatory proteins identified in *A. variegatum* saliva.

**UniProt protein IDs (Leading protein)**	**Description**	**Organism**	**Number of proteins**	**Peptides**	**Unique peptides**	**Sequence coverage [%]**	**Mol. weight [kDa]**	**Propsearch distance (reliability)**	**Propsearch putative protein family**
**NUCLEIC-ACID BINDING PROTEINS**									
F0J9Z3	RAS oncogene family member RAB1	*A. variegatum*	5	1	1	11	16.156	5.65–7.34 (99.6%)	GTP-binding protein
**ANTI-OXYDANT ENZYMES**									
A0A023G0Q2	Putative his-rich 1	*A. parvum*	2	2	2	14.9	15.48	1.08–2.02 (99.9 > x > 99.6%)	Superoxide dismutase [Mn-Fe] (EC 1.15.1.1) (Fragment)
G3MKP9	Carboxypeptidase	*A. maculatum*	2	1	1	2.6	52.321	5.60 (99.6%)	Dopachrome tautomerase precursor (EC 5.3.3.12) (DT) (DCT)
**RIBOSOMAL PROTEINS**									
G3MMA6	Putative uncharacterized protein (Putative alpha crystallins?)	*A. maculatum*	8	1	1	6	28.586	3.77–7.18 (99.6%)	60S ribosomal protein L3
**UBIQUITIN COMPLEX**									
A0A023GMF4	Putative beta-spectrin (Fragment)	*A. triste*	2	1	1	0.8	205.72	5.14–5.44 (99.6%)	Spectrin alpha chain

## Discussion

In the present study, we demonstrated high immunomodulation of bovine immune cells, lymphocytes and macrophages by *A. variegatum* saliva. This adds a new tick species to the increasing reports on immunomodulation by tick saliva (Kazimírová and Štibrániová, 2013). Increasing data from the literature on the interactions between tick saliva and the cells of the innate and adaptive immune system of vertebrate hosts tend to demonstrate that excreted-secreted salivary factors are critical both for completing the tick developmental cycle via blood feeding and for the transmission/reactivation of pathogens (Kotál et al., 2015; Šimo et al., 2017). Here, we showed that *A. variegatum* saliva induced inhibition of ConA-induced proliferation of bovine lymphocytes in a dose-dependent manner. This immunosuppression effect has already been observed in different tick models, and with various mitogens, as reviewed by Wikel and Kazimírová (Kazimírová and Štibrániová, 2013; Wikel, 2013). For example, *Ixodes ricinus* salivary glands secreted a protein that suppressed T lymphocyte proliferation and inhibited macrophage pro-inflammatory cytokines (Kovár et al., 2001, 2002). Among *Amblyomma* ticks, *A. cajennense* saliva has also been reported to inhibit lymphocyte proliferation in mouse and horse (Castagnolli et al., 2008). Another study showed that *Dermacentor andersoni* gland extract inhibited the pro-inflammatory cytokine TNF-α and up-regulated the anti-inflammatory cytokine IL-10, with down regulation of the expression of MHC II and of co-stimulation molecules (Wikel, 2013). These effects of saliva from different tick species, associated with our similar findings on *A. variegatum* saliva, strengthen the hypothesis that tick-host interactions generally result in induced lymphocyte anergy and reduced macrophage antigen presenting capacity and the classical activation pathway. In our study, we also showed that *A. variegatum* saliva emphasized the macrophage LPS-induced down regulation of CMH II, decreased the LPS-induced up regulation of CD40 and CD80. While CD40 binds on CD40-L on T cells for activation, inhibition of the expression of this molecule might affect directly the proliferative response of T-cells, as we observed for ConA-stimulated PBMC subjected to saliva. Similarly, while CD80 favors a Th1 response, the inhibition of the expression of LPS-induced CD80 might switch toward a Th2 response of lymphocytes, switching to an anti-inflammatory response. This switch has been demonstrated to be induced by *I. ricinus* salivary gland extracts (Kovár et al., 2001, 2002), and plays a role in countering the Th1-oriented response following tick aggression, as demonstrated for *Rhipichephalus microplus* in bovines (Brake and Pérez de León, 2012). This effect could result in the global inhibition of antigen presentation capacities and pro-inflammatory activation pathway of these immune cells, even if they are activated by the pathogen at the biting site. Furthermore, we evidenced that *A. variegatum* saliva also had an effect on NO production by macrophages through complex modulation of this microbicidal and immunosuppressive molecule (Wink et al., 2011): it increased its production at relatively low concentrations but decreased its production at high concentrations. Macrophages use NO together with several other free radicals as a mechanism for killing pathogens and inducing an inflammatory response, but high levels of NO has been shown to have suppressive effects on lymphocyte proliferation (MacMicking et al., 1997). We observed that NO production was increased by small quantities of saliva (31.25–62.5 μg/ml), then strongly decreased by high concentrations of saliva (up to 250 μg/ml). This inhibition correlated with the decrease in TNF-α and the increase in IL-10 we observed when the global *in vitro* immune response became clearly anti-inflammatory. It is possible that high production of NO plays a role in the establishment of lymphocyte anergy, before the deactivation of macrophages by *A. variegatum* saliva avoids tissue damage in the host that would be deleterious to tick feeding. In a previous study on *A. variegatum* saliva performed in the *in vivo* mouse model, we showed that, in skin lesions mimicking tick biting, *A. variegatum* saliva impairs leukocyte infiltration (Vachiery et al., 2015). Experiments in an *in vitro* human model further demonstrated that tick saliva impaired the migration of antigen presenting cells to the draining lymph nodes and affected the recruitment of blood monocytes to the skin lesion and prevented their maturation *in situ* (Vachiery et al., 2015). These results strengthen our findings with bovine cells, and show how the saliva of *A. variegatum*, by modulating chemotaxis, the proliferative capacity of lymphocytes, the regulatory capacities of macrophages, diverts the host's immune system to its own advantage, and the consequences this may have for the transmission or reactivation of pathogens.

Some of the strong and specific effects of tick saliva in modulating cytokine production by macrophage and lymphocytes, and hence the impacts on immune response, can be analyzed at molecular scale to characterize saliva molecules thanks to the advent of proteomics. Among the proteins we evidenced in *A. variegatum* female saliva and shown to have direct immunomodulatory functions, the CAP superfamily of proteins (comprising the CRISP, Antigen-5, and pathogen-related-1 families) has been found in most tick sialomes studied to date, with molecular characteristics similar to wasp-venom proteins and annotated as Antigen-5 (Gibbs et al., 2008). Additionally, recombinant Da-p36 has been demonstrated to suppress T-lymphocyte-mitogen-driven *in vitro* proliferation of splenocytes (Alarcon-Chaidez et al., 2003), and could therefore be part of the molecular mechanism behind our observation of inhibition lymphocyte proliferation in the presence of *A. variegatum* saliva.

The most representative category of proteins with potential immunomodulatory properties we identified were protease inhibitors, mainly serpins. Serpins are abundant in ticks, and one of their functions is to modulate the host immune system (Chmelar et al., 2017). The first tick serpin described for its effect on host defense mechanisms was named *I. ricinus* immunosuppressor (Iris) and was able to inhibit T-cell and splenocyte proliferation and to modify cytokine levels derived from PBMC (Leboulle et al., 2002), as well as to bind to monocytes/macrophages and to suppress TNF-α secretion (Prevot et al., 2009). These results are very similar to our observations, and we hypothesize the existence of an Iris-like protein among the serpins identified in *A. variegatum* saliva. Other protease inhibitors we identified are cysteine protease inhibitors named thyropins. Recently, an exogenous thyropin was shown to affect IL-12 secretion of dendritic cells (DCs) (Zavašnik-Bergant and Bergant Marušič, 2016), which leads us think that the thyropins in *A. variegatum* saliva could be involved in the decreased production of IL-12 by bovine macrophages, like DCs. In contrast to protease inhibitors, we identified proteases in the saliva of *A. variegatum*, whose carboxypeptidases could play a role in the destruction of inflammatory peptide agonists (Ribeiro et al., 2011) and cathepsin-like cysteine proteases could destroy cellular mediators of inflammation (Radulović et al., 2014).

During tick feeding, the processing of the blood meal is accompanied by a high risk of oxidative stress linked to large amounts of heme, a bi-product of hemoglobin digestion, and free iron in the host's blood (Graca-Souza et al., 2006). We identified ferritin and hemelipoproteins in *A. variegatum* saliva, which could be used to dump heme and free iron in the host to avoid oxidative stress, and at the same time benefit tick-borne pathogens that may need iron to proliferate (Rouault, 2004). We also found numerous anti-oxidant enzymes (including glutathione-S transferase (GST), protein disulfide isomerase, thioredoxin peroxidase, peroxidasin, superoxide dismutase, and selenoproteins) in the saliva of *A. variegatum*, which could play a role in reducing the toxicity of reactive oxygen and nitrogen species (such as NO) produced as part of the wound healing mechanism and anti-microbial defenses (Vider et al., 2001; Rojkind et al., 2002). Preventing damage caused by oxidative stress could benefit both the host and the tick, but given the susceptibility of pathogens to the products of oxidative stress, anti-oxidants in tick saliva could facilitate the transmission of tick-borne pathogens. Interestingly, we found D-dopachrome tautomerase, the functional homolog of macrophage migration inhibitory factor (MIF) (Merk et al., 2011), which is a pro-inflammatory cytokine produced by ticks to regulate host responses to tick feeding (Wasala et al., 2012). D-dopachrome tautomerase/MIF could be involved in the immunological silence induced by *A. variegatum*, with no chemotactic recruitment of host macrophages. Conversly, we evidenced several proteins dedicated to anti-inflammatory response. For instance, extracellular nucleic acids are potent proinflammatory molecules, and nucleic acid binding proteins could be part of the system used by the tick to modulate host inflammation (Radulović et al., 2014). In the same way, ribosomal and heat shock proteins have been described as potent anti-inflammatory molecules (Pockley, 2003; Poddar et al., 2013).

Fatty acid and histamine binding proteins also play roles in preventing inflammation during tick feeding. Histamine is a potent pro-inflammatory molecule that is released by cellular mediators of inflammation such as mast cells and neutrophils (White and Kaliner, 1987). Like *A. americanum, A. variegatum* saliva contains histamine-binding protein/lipocalin to sequester histamine and stop the corresponding inflammatory response (Radulović et al., 2014). Although other authors have demonstrated secretion of prostaglandin E2 (PGE2) and prostacyclin (PGI) in tick saliva (Ribeiro et al., 1988; Bowman et al., 1995), we did not identify these proteins among our proteomics data. However, we did identify a phospholipase A2, which could stimulate prostacyclin production (Bowman et al., 1997) and induce PGE2 production by host cells (Hara et al., 1991; Miyake et al., 1994). Tick saliva PGE2 has been shown to modulate the function of macrophages, particularly cytokine profiles (Poole et al., 2013), as we also evidenced in our study. Indirect PGI and PGE2 regulation could have been developed by *A. variegatum* to manipulate host macrophages, which, as a central immune cell, could pervert the whole immune system of the host.

Multifunctional calcium binding proteins, such as calponin and calreticulin (CRT), are present in the saliva of *A. variegatum* but apart from depleting Ca^2+^ to prevent activation of blood clotting (Astrup, 2009), their function is not yet known. Nevertheless, an interesting hypothesis has emerged from *A. americanum* CRT, which could skew a Th-2 host response to tick feeding (Kim et al., 2015), and which is consistent with our observation of a M2-induced profile of macrophage cytokines/metabolism by *A. variegatum* saliva.

Finally, we also discovered an original ubiquitination complex in the saliva of *A. variegatum*, both on its molecular architecture and on its extracellular localization. In eukaryote cells, ubiquitin is transferred by three enzymes: the ubiquitin-activating enzyme E1 ligase catches ubiquitin and transfers it to the ubiquitin-conjugating enzyme E2 ligase, which associates with ubiquitin-ligating enzyme E3 ligase to transfer ubiquitin to the target substrate (Tanaka et al., 1998). Intriguingly, the ubiquitination we evidenced in *A. variegatum* is simplified with spectrin playing the role of a chemeric E2/E3 ubiquitin-conjugating-ligating enzyme (Goodman et al., 2015). Whereas, ubiquitination has long been well described in connection with proteasome degradation of proteins, in the last decade, an increasing literature has proposed an important role for ubiquitination in immunity (Ben-Neriah, 2002; Zinngrebe et al., 2014), particularly in regulating both innate and adaptive immune responses (Malynn and Ma, 2010; Park et al., 2014; Hu and Sun, 2016). Interestingly, extracellular ubiquitin has been implicated in lymphocyte differentiation, suppression of the immune response, and prevention of inflammation (Sujashvili, 2016), particularly by binding to the CXCR4 receptor (Majetschak, 2011; Scofield et al., 2015). Moreover, extracellular ubiquitin can bind directly to regulatory T cells; enhancing their inhibitory effect on effector T cells proliferation (Cao et al., 2014). Exogenous ubiquitin can also reduce TNF-α production (Majetschak et al., 2004). The last two elements are in complete agreement with our observations on inhibition of lymphocyte proliferation and reduction of TNF-α production by macrophages in the presence of tick saliva, and make us suggest that *A. variegatum* uses its extracellular ubiquitination complex as an effective molecular weapon for render the immune responses of the host ineffective during the blood meal.

**Figure 5:**
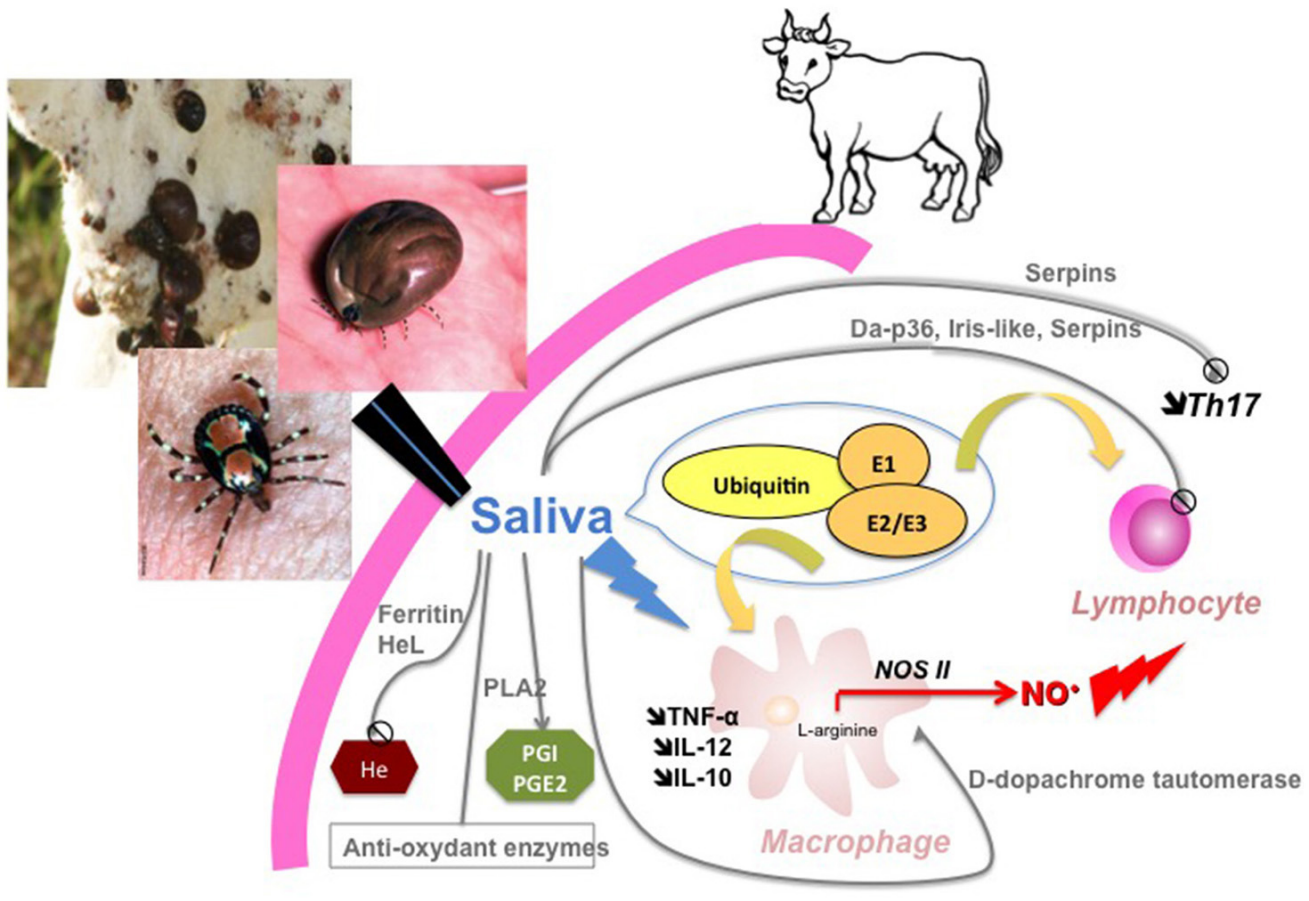
Synthetic view of *A. variegatum* salivary-induced modulation of bovine immune cells Molecular determinants (with immunomodulatory properties identified by proteomics) driving cellular events (inhibition of lymphocyte proliferation, modulation of macrophage activation) evidenced in the study are summarized in the scheme.

To conclude, our data shed new light on the molecular determinants of *A. variegatum* saliva-induced immunomodulation of bovine immunity cells and allows us to propose a new synthesis scheme of the cellular and molecular events that take place at the tick-host interface (Figure 5). Further studies on the effect of *A. variegatum* saliva on bovine PBMC and macrophages are needed to better understand the mechanisms underlying our observations, particularly the comparative effect of saliva on different lymphocyte sub-populations such as cytotoxic T cells or NK cells, but also qualitative or quantitative cytokine modulation such as INF-γ, IL-2, IL-4, or IL-17 with impacts on Th1/Th2 or Th17 balance, as shown in *I. ricinus* (Kopecký et al., 1999; Kovár et al., 2001; Leboulle et al., 2002) and in mosquitoes (Wanasen et al., 2004). Effects on macrophages presenting capacities also need to be elucidated in connection with pathogen uptake and presentation in the presence of tick saliva. These cellular investigations will need to be combined with further molecular characterization of saliva proteins to identify the key molecules in immunomodulation mechanisms. Overall, our data provide new insights into tick-ruminant interactions for *A. variegatum*, including their involvement in vectorial competence for pathogen transmission (*E. ruminantium* infection) or on pathogen reactivation (dermatophilosis), and pave the way for integrative strategies to interfere with both the immunosuppressive and infectious processes in the corresponding tick-borne diseases.

## Author contributions

VR, BF, FS, and PH: designed the work; VR, AV, LC, RA, KG-G, and PH: generated the biological samples; VR, BF, AV, LC, AA, RA, KG-G, OV, ED, MS, and PH: performed the experiments; VR, BF, AV, LC, AA, OV, ED, MS, FS, EL, NV, and PH: acquired, analyzed, and interpreted the data; VR and PH: wrote the manuscript; VR, BF, AV, LC, AA, OV, ED, MS, RA, KG-G, FS, EL, NV, and PH: revised and approved the final version of the manuscript.

### Conflict of interest statement

The authors declare that the research was conducted in the absence of any commercial or financial relationships that could be construed as a potential conflict of interest.

## Supplementary material

Supplementary Figure 1 Effect of *A. variegatum* saliva on the expression of surface molecules of bovine unstimulated macrophages. Bovine blood monocyte-derived macrophages were cultivated for 24 h with different concentrations of *A. variegatum* saliva. Cells were collected and stained for MHC II, CD40, CD80, and CD86 surface markers. Expression levels (mean of fluorescence intensity, MFI) of markers on macrophages compared to those on control unstimulated cells were measured by flow cytometry. Blue lines and shading show the linear regression fit and the 0.95 confidence interval, respectively. The *p*-value of the model is indicated on the panel.

Supplementary Figure 2 Effect of *A. variegatum* saliva on the production of cytokines by bovine unstimulated macrophages. Bovine blood monocyte-derived macrophages were stimulated for 24 h with different concentrations of *A. variegatum* saliva. Cell culture supernatants were collected and IL-10, IL-12, and TNF-α production was titrated by ELISA. (Data are results obtained for one experiment performed on three different animals; note different scales on graphs). Blue lines and shading show the linear regression fit and the 0.95 confidence interval, respectively. The *p*-value of the model is indicated on the panel.
