# Supplementary figures and images for: Immunomodulatory Effects of Amblyomma variegatum Saliva on Bovine Cells: Characterization of Cellular Responses and Identification of Molecular Determinants
Source: Front Cell Infect Microbiol. 2018 Jan 4;7:521. doi: 10.3389/fcimb.2017.00521 (PMC5759025; doi:10.3389/fcimb.2017.00521)

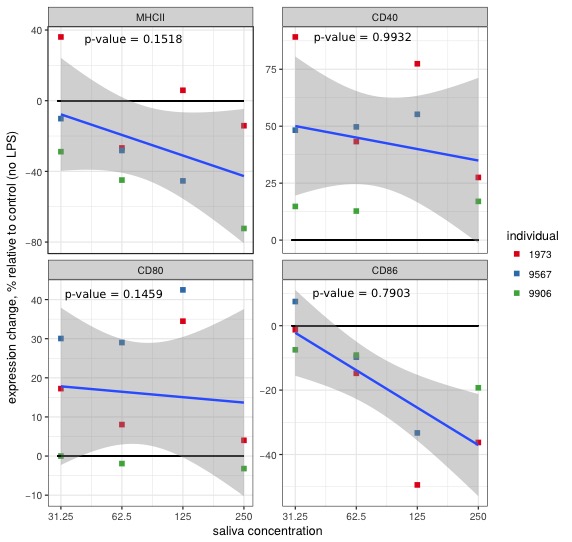

Supplement: Supplementary Figure 1 — Effect of A. variegatum saliva on the expression of surface molecules of bovine unstimulated macrophages. Bovine blood monocyte-derived macrophages were cultivated for 24 h with different concentrations of A. variegatum saliva. Cells were collected and stained for MHC II, CD40, CD80, and CD86 surface markers. Expression levels (mean of fluorescence intensity, MFI) of markers on macrophages compared to those on control unstimulated cells were measured by flow cytometry. Blue lines and shading show the linear regression fit and the 0.95 confidence interval, respectively. The p-value of the model is indicated on the panel. [file Image1.jpeg]

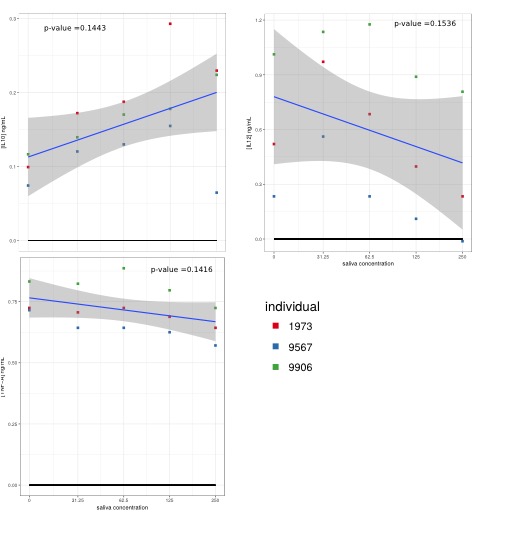

Supplement: Supplementary Figure 2 — Effect of A. variegatum saliva on the production of cytokines by bovine unstimulated macrophages. Bovine blood monocyte-derived macrophages were stimulated for 24 h with different concentrations of A. variegatum saliva. Cell culture supernatants were collected and IL-10, IL-12, and TNF-α production was titrated by ELISA. (Data are results obtained for one experiment performed on three different animals; note different scales on graphs). Blue lines and shading show the linear regression fit and the 0.95 confidence interval, respectively. The p-value of the model is indicated on the panel. [file Image2.jpeg]
